# The Diagnostic Roles of Cytokines in Hepatobiliary Cancers

**DOI:** 10.1155/2017/2979307

**Published:** 2017-12-19

**Authors:** Yavuz Savas Koca, Mahmut Bulbul, Ibrahim Barut

**Affiliations:** ^1^Department of General Surgery, School of Medicine, Suleyman Demirel University, Isparta, Turkey; ^2^HPB Surgery Unit, Department of General Surgery, School of Medicine, Suleyman Demirel University, Isparta, Turkey

## Abstract

**Objectives:**

The aim of this study was to investigate the role of several cytokines including IL-2, IL-6, IL-8, IL-10, and TNF-*α* in the diagnosis of HPB cancers.

**Materials and Methods:**

The prospective study was performed between October 2007 and September 2014. The study included 226 patients who were divided into 5 groups depending on their postoperative and histopathologic diagnosis: Control group included 30 healthy volunteers. Hepatocellular cancer (HCC) group included 24 patients diagnosed with HCC. Gallbladder cancer (GBC) group included 36 patients diagnosed with GBC. Cholangiocellular carcinoma group included 64 patients diagnosed with cholangiocellular carcinoma. Pancreatic cancer group included 72 patients diagnosed with pancreatic cancer. Serum levels of IL-2, IL-6, IL-8, IL-10, and TNF-*α* were measured using an enzyme-linked immunosorbent assay kit in accordance with the guidelines of the producer.

**Results:**

Serum TNF-*α* concentration was significantly higher in the cholangiocellular carcinoma and pancreatic cancer groups compared to other groups. IL-6 and IL-10 were significantly increased in both the HCC and GBC groups, IL-2, IL-6, IL-10, and TNF-*α* in the cholangiocellular carcinoma group, and IL-2, IL-6, IL-8, and TNF-*α* in the pancreatic cancer group.

**Conclusion:**

We suggest that cytokines can be used as useful markers in the diagnosis of HPB cancers.

## 1. Introduction

Hepatopancreatobiliary (HPB) cancers, which are becoming more and more common due to the increasing levels of industrialization and smoking, are often diagnosed at late stages and thus do not allow curative treatment [1]. Moreover, HPB cancers do not allow surgical resection due to their localization and proximity to vital organs; therefore, they are mostly accepted as late-stage cancers when diagnosed. Early diagnosis of HPB cancers has vital importance for the effective management and treatment of these cancers [2].

HPB system includes liver, intra- and extrahepatic bile ducts, gallbladder, and pancreas. Response to surgery, external factors, infectious traumas, and tumors are characterized by various metabolic, endocrinal, and immunological alterations in patients [3]. Tumor immunology is the study of the interaction between cancer and the immune system. The primary fields of tumor immunology include the response mechanisms against the initiation and the progression of tumors, the evasive techniques used by the tumors to avoid the immune system, and the diagnosis and treatment of tumors via immunological techniques [4].

Although all the immune cells play a role in the destruction of tumor cells, cellular immunity plays the key role in this process. In cellular immunity, helper T-lymphocytes play a role via the lymphokines they secrete (interleukins, interferon, and tumor necrosis factor-beta (TNF-*β*)), whereas macrophages play a role via the reactive oxygen metabolites, and nitric oxide plays a role by means of various enzymes and lymphokines such as TNF-*α* [4].

Cytokines are molecules produced by immune cells that have local and systemic effects. Cytokines play a dramatic role in tumor immunity. The most well-known cytokines include interleukin-2 (IL-2), interferons, granulocyte macrophage-colony stimulating factor (GM-CSF), TNF, tumor growth factor (TGF), IL-10, and prostaglandin E2 (PGE2) [5].

In this study, the role of several cytokines including IL-2, IL-6, IL-8, IL-10, and TNF-*α* in the diagnosis of HPB cancers was investigated.

## 2. Material and Method

Group I (control group) included 30 healthy volunteers who were either students or research assistants at our Medical School. The blood samples of the female participants were obtained when they were outside their menstruation period. Systemic physical examination was performed in all the patients and no signs of infection were detected in any patient. The study included 196 patients who were divided into 4 groups depending on histopathologic diagnosis: Group II (hepatocellular cancer (HCC) group) included 24 patients diagnosed with HCC. Group III (gallbladder cancer (GBC) group) included 36 patients diagnosed with GBC. Group IV (cholangiocellular carcinoma group) included 64 patients diagnosed with cholangiocellular carcinoma. Group V (pancreatic cancer group) included 72 patients diagnosed with pancreatic cancer. Cytokine levels may be increased in all inflammatory processes [4]. To investigate whether this increase is associated with inflammation or tumor and to analyze the diagnostic use of cytokines, the patients who were diagnosed with HPB cancer and had a total bilirubin level of <2 g/dl and a CRP level of <3 were included in the study.

A blood sample (2-3 cc) was obtained from each participant, and serum was separated and stored in a freezer after the centrifuging process. The serum samples were analyzed using an enzyme-linked immunosorbent assay (ELISA) kit in accordance with the guidelines of the producer. Serum levels of IL-2, IL-6, IL-8, IL-10, and TNF-*α* were measured and expressed as pg/ml.

Statistical analyses were performed by using SPSS 15.0 for Windows (Chicago, IL, USA). The variables were analyzed using the Kruskal Wallis test, Mann–Whitney *U* test, *t*-test, chi-square test, and one-way ANOVA. A *p* value of <0.05 was considered significant.

Informed consent was obtained from the patients.

## 3. Results

The study included 226 participants, 134 (59.3%) males and 92 (40.7%) females, with a mean age of 49.89 (range, 22–92 years) years.

The cytokine concentrations measured in the control, HCC, GBC, cholangiocellular carcinoma, and pancreatic cancer groups are presented in Tables 1, 2, 3, 4, and 5, respectively.

**Figure 1:**
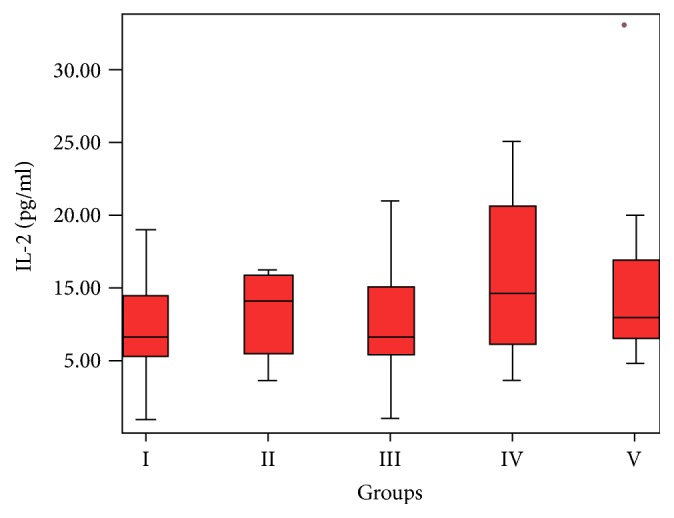
Increased serum IL-2 concentration in the cholangiocellular carcinoma group (Group IV) (*p* = 0.018).

Serum IL-2 concentration was significantly higher in the GBC group compared to the control group (in both one-way ANOVA + LSD test (*p* = 0.018) and *t*-test (*p* < 0.001)) (Figure 1).

**Figure 2:**
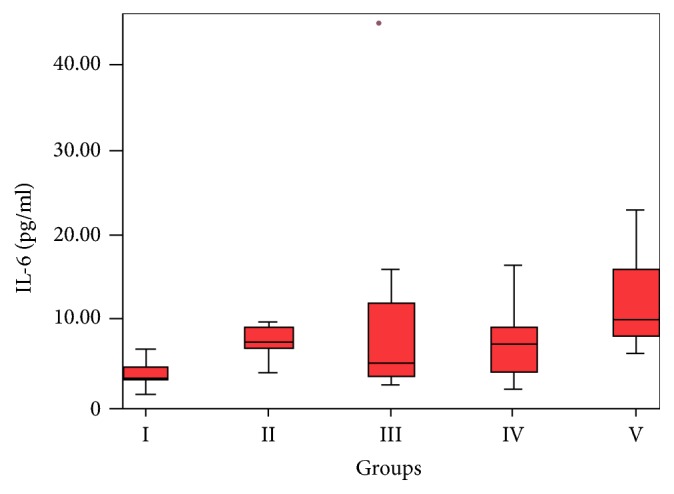
Increased serum IL-6 concentrations in the GBC and pancreatic cancer groups (Groups III and V) (*p* = 0.034 and *p* ≤ 0.001, resp.).

Serum IL-6 concentration was significantly higher in the GBC and pancreatic cancer groups compared to the control group in one-way ANOVA + Tukey's test (*p* = 0.034 and *p* < 0.001, resp.) (Figure 2); however, no significant difference was found between the groups (*p* = 0.520). Similarly, serum IL-6 concentration was significantly higher in the HCC and cholangiocellular carcinoma groups compared to the control group (*p* = 0.006 and *p* = 0.003, resp.). These findings indicate that serum IL-6 concentration was significantly increased in all of the patients with HPB cancers.

**Figure 3:**
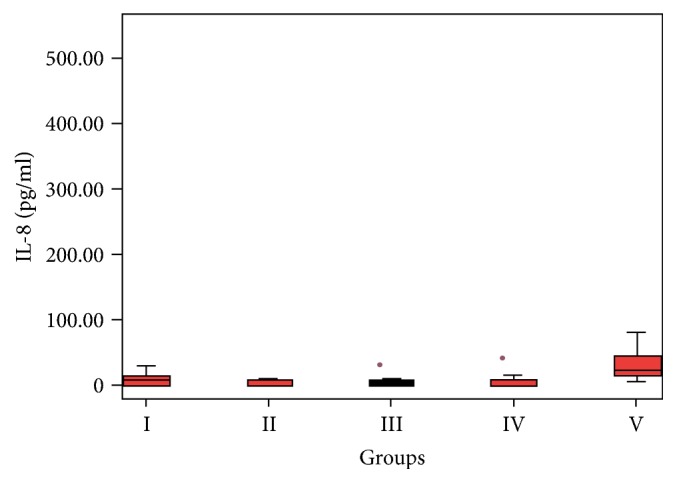
Significantly increased IL-8 concentration in the pancreatic group (*p* = 0.009).

Serum IL-8 concentration was significantly increased only in the pancreatic group (*p* = 0.009) (Figure 3).

**Figure 4:**
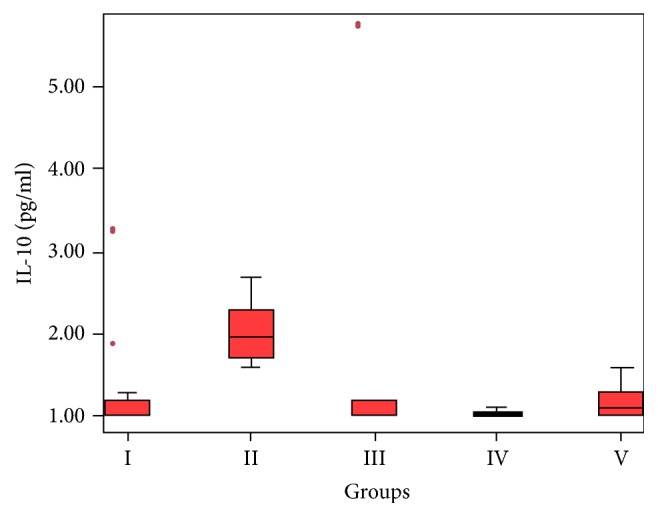
Significantly increased serum IL-10 concentration in the HCC group (*p* = 0.003).

Serum IL-10 concentration was significantly increased in the HCC group (*p* = 0.003) (Figure 4). Similarly, serum IL-10 concentration was higher in the GBC group compared to the control group but no significant difference was observed (*p* = 0.078). However, the HCC group established a significant difference with all of the groups except for the GBC group (*p* = 0.187). In addition, the GBC group established a significant difference with the control group (*p* = 0.013).

**Figure 5:**
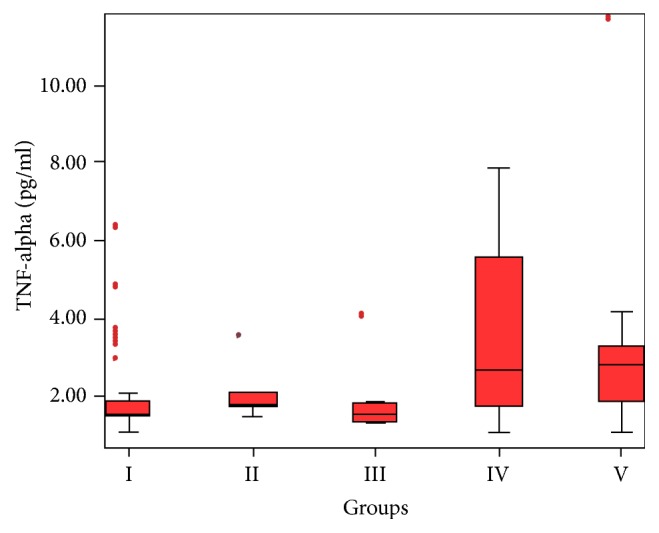
Significantly increased serum TNF-*α* concentration in the cholangiocellular carcinoma and pancreatic cancer groups (*p* = 0.007 and *p* = 0.044).

Serum TNF-*α* concentration was significantly higher in the cholangiocellular carcinoma and pancreatic cancer groups compared to other groups (*p* = 0.007 and *p* = 0.044) (Figure 5); however, no significant difference was observed (*p* = 0.473).

## 4. Discussion

The results indicated that the cytokine concentrations were significantly increased in the patients with hepatopancreatobiliary (HPB) cancers. IL-6 and IL-10 were significantly increased in both the HCC and GBC groups, whereas IL-2, IL-6, IL-10, and TNF-*α* were significantly increased in the cholangiocellular carcinoma group, and IL-2, IL-6, IL-8, and TNF-*α* were significantly increased in the pancreatic cancer group.

The primary function of the immune system is to protect the organism against bacterial and viral infections and to minimize the injury in the cells and tissues infected by microorganisms. The regulatory T (Treg) cells play a key role in the achievement of this function [3, 6].

The levels of Treg cells such as CD4^+^ and CD25^+^ have been shown to be increased in lung, pancreatic, breast, liver, and skin cancers [6–9]. Some of the chemokines and cytokines secreted by these cells stimulate chemotaxis and immunity [3].

IL-2 is a lymphokine which is secreted from activated T cells and generates effective cytotoxic response [10]. Studies have shown that decreased IL-2 concentration leads to lower survival rates [11, 12]. Therefore, in several studies, IL-2 therapy has been performed to increase the survival rates [10]. In our study, IL-2 concentration was increased in patients with cholangiocellular carcinoma and pancreatic cancer. However, it cannot be asserted that these concentrations correspond to increased survival rates because further large-scale studies with larger patient series are needed to investigate the relationship between IL-2 concentration and survival. Nevertheless, the increased IL-2 concentrations found in this study may be used as biomarkers in early diagnosis of HPB cancers.

IL-6 is an inflammatory and pleiotropic cytokine [13]. This cytokine is secreted from T cells, macrophages, and stromal cells through the mediation of TNF-*α* and IL-1 [14, 15]. Increased IL-6 concentrations have been reported in numerous diseases such as cardiovascular diseases, Type 2 diabetes, and lymphoma, kidney, bladder, and colorectal cancers [16]. In our study, IL-6 concentration was increased in all of the study groups compared to the control group, suggesting that IL-6 concentration was increased in all the patients with HPB cancers. Although this finding decreases the specificity, it is highly important. However, as mentioned above, IL-6 concentration may also increase in several nonneoplastic diseases. In our study, since the tumor groups were compared with healthy controls, the significant increase in IL-6 concentration may be a useful marker for HPB cancers. Depending on the fact that IL-6 is secreted through the mediation of TNF-*α* and IL-1 and on the finding that IL-6 concentration was increased in our HCC and GBC groups, we consider that IL-6 may be a useful marker for HCC and GBC. Moreover, the finding that indicated increased IL-6 concentration in the cholangiocellular carcinoma group is consistent with the findings of the studies which reported that IL-6 may be used as a marker of cholangiocellular carcinoma and IL-6 can be used in anticancer chemotherapy [17, 18].

IL-8 concentration was significantly increased only in the pancreatic group, which is consistent with the literature [19]. IL-8 and IL-1 have been shown to be indicators of metastatic pancreatic cancer. As suggested by Matsuo et al. [20], we also consider that IL-8 not only is as a cytokine whose concentration is increased only in metastatic pancreatic cancer but also is a special marker of pancreatic cancer.

IL-10 concentration was significantly increased in the HCC, GBC, and cholangiocellular carcinoma groups. Although IL-10 concentration has been previously shown to be increased in liver and lung cancers [21, 22], there is no documentation of increased IL-10 concentrations in GBC and cholangiocellular carcinoma. Therefore, this study provides significant contribution to the literature since it is the first study to report on increased IL-10 concentrations in GBC and cholangiocellular carcinoma.

TNF-*α* concentration was increased in the cholangiocellular carcinoma and pancreatic cancer groups. Egberts et al. [23] suggested that the use of anti-TNF-*α* therapy in pancreatic cancer may prevent metastasis and provide antitumor activity. Similarly, Utaisincharoen et al. [24] reported that anti-TNF-*α* therapy should be the method of choice in the treatment of cholangiocellular cancer. Nevertheless, these findings contradict our findings since we found increased TNF-*α* concentrations in the patients with cholangiocellular cancer. Therefore, further large-scale studies are needed to substantiate these findings.

In conclusion, cytokines can be used as useful markers in the diagnosis of HPB cancers.

## Figures and Tables

**Table 1:** Cytokine concentrations measured in the control group.

Control group (*N* = 30)	Mean	Median	SD	Min–max
Age	23.87	23.00	2.25	22–30
IL-2	7.44	6.70	3.17	1.20–14.20
IL-6	3.48	3.20	1.37	1.10–6.60
IL-8	11.33	10.25	7.70	1.30–32.60
IL-10	1.20	1.00	0.44	1.00–3.20
TNF-*α*	2.03	1.60	1.13	1.10–6.20

SD: standard deviation.

**Table 2:** Cytokine concentrations measured in the hepatocellular cancer (HCC) group.

HCC group (*N* = 24)	Mean	Median	SD	Min–max
Age	68.83	72.50	10.26	52–79
IL-2	8.36	9.29	3.12	3.70–11.30
IL-6	7.28	7.47	2.15	3.70–9.70
IL-8	8.63	9.45	3.88	3.60–14.00
IL-10	2.03	1.95	0.41	1.60–2.70
TNF-*α*	2.03	1.80	0.65	1.50–3.30

SD: standard deviation.

**Table 3:** Cytokine concentrations measured in the gallbladder cancer (GBC) group.

GBC group (*N* = 36)	Mean	Median	SD	Min–max
Age	66.56	68.00	11.13	44–82
IL-2	7.72	6.70	4.36	1.20–16.03
IL-6	10.48	4.80	13.60	2.23–44.53
IL-8	8.48	6.59	8.13	1.30–26.94
IL-10	1.61	1.00	1.54	1.00–5.70
TNF-*α*	1.83	1.60	0.81	1.27–3.92

SD: standard deviation.

**Table 4:** Cytokine concentrations measured in the cholangiocellular carcinoma (CC) group.

CC group (*N* = 64)	Mean	Median	SD	Min–max
Age	67.50	68.50	12.30	36–92
IL-2	10.79	9.63	5.60	3.80–20.13
IL-6	7.07	7.05	4.00	1.80–16.41
IL-8	7.72	4.47	8.70	1.30–36.33
IL-10	1.03	1.00	0.06	1.00–1.20
TNF-*α*	3.56	2.70	2.62	1.06–7.81

SD: standard deviation.

**Table 5:** Cytokine concentrations measured in the pancreatic cancer group.

Pancreatic cancer group (*N* = 72)	Mean	Median	SD	Min–max
Age	62.94	63.00	13.96	38–84
IL-2	10.14	8.10	5.44	5.02–27.79
IL-6	13.18	9.95	7.99	5.94–38.10
IL-8	58.71	21.81	123.57	8.72–546.44
IL-10	1.17	1.10	0.22	1.00–1.80
TNF-*α*	3.12	2.85	2.26	1.05–11.42

SD: standard deviation.

## References

[1] Burak K., Angulo P., Pasha T. M., Egan K., Petz J., Lindor K. D. (2004). Incidence and Risk Factors for Cholangiocarcinoma in Primary Sclerosing Cholangitis. *American Journal of Gastroenterology*.

[2] Abbas G., Lindor K. D. (2009). Cholangiocarcinoma in primary sclerosing cholangitis. *Journal of Gastrointestinal Cancer*.

[3] Roshani R., McCarthy F., Hagemann T. (2014). Inflammatory cytokines in human pancreatic cancer. *Cancer Letters*.

[4] Slingluff C. L., Norton J. A. (2001). Immunology of cancer. *Surgery; Basic Science and Clinical Evidence*.

[5] Cools N., Ponsaerts P., Van Tendeloo V. F. I., Berneman Z. N. (2007). Regulatory T cells and human disease. *Clinical and Developmental Immunology*.

[6] Wolf A. M., Wolf D., Steurer M., Gastl G., Gunsilius E., Grubeck-Loebenstein B. (2003). Increase of regulatory T cells in the peripheral blood of cancer patients. *Clinical Cancer Research*.

[7] Barut I., Kaya S. (2014). The diagnostic value of C-reactive protein in bacterial translocation in experimental biliary obstruction. *Advances in Clinical and Experimental Medicine*.

[8] Liyanage U. K., Moore T. T., Joo H.-G. (2002). Prevalence of regulatory T cells is increased in peripheral blood and tumor microenvironment of patients with pancreas or breast adenocarcinoma. *The Journal of Immunology*.

[9] Ormandy L., Hillemann T., Wedemeyer H., Manns M. P., Greten T. F., Korangy F. (2005). Increased populations of regulatory T cells in peripheral blood of patients with hepatocellular carcinoma. *Cancer Research*.

[10] Grande C., Firvida J. L., Navas V., Casal J. (2006). Interleukin-2 for the treatment of solid tumors other than melanoma and renal cell carcinoma. *Anti-Cancer Drugs*.

[11] Dempe S., Lavie M., Struyf S. (2012). Antitumoral activity of parvovirus-mediated IL-2 and MCP-3/CCL7 delivery into human pancreatic cancer: Implication of leucocyte recruitment. *Cancer Immunology, Immunotherapy*.

[12] Xiao Z., Luo G., Liu C. (2014). Molecular mechanism underlying lymphatic metastasis in pancreatic cancer. *BioMed Research International*.

[13] Heikkilä K., Ebrahim S., Lawlor D. A. (2008). Systematic review of the association between circulating interleukin-6 (IL-6) and cancer. *European Journal of Cancer*.

[14] Hodge D. R., Hurt E. M., Farrar W. L. (2005). The role of IL-6 and STAT3 in inflammation and cancer. *European Journal of Cancer*.

[15] Song M., Kellum J. A. (2005). Interleukin-6. *Critical Care Medicine*.

[16] Aggarwal B. B., Shishodia S., Sandur S. K., Pandey M. K., Sethi G. (2006). Inflammation and cancer: how hot is the link?. *Biochemical Pharmacology*.

[17] Holmer R., Goumas F. A., Waetzig G. H., Rose-John S., Kalthoff H. (2014). Interleukin-6: A villain in the drama of pancreatic cancer development and progression. *Hepatobiliary & Pancreatic Diseases International*.

[18] Mott J. L., Gores G. J. (2007). Targeting IL-6 in cholangiocarcinoma therapy. *American Journal of Gastroenterology*.

[19] Delitto D., Black B. S., Sorenson H. L. (2015). The inflammatory milieu within the pancreatic cancer microenvironment correlates with clinicopathologic parameters, chemoresistance and survival. *BMC Cancer*.

[20] Matsuo Y., Sawai H., Funahashi H. (2004). Enhanced Angiogenesis Due to Inflammatory Cytokines from Pancreatic Cancer Cell Lines and Relation to Metastatic Potential. *Pancreas*.

[21] Hsia C.-Y., Huo T.-I., Chiang S.-Y. (2007). Evaluation of interleukin-6, interleukin-10 and human hepatocyte growth factor as tumor markers for hepatocellular carcinoma. *European Journal of Surgical Oncology*.

[22] Karakhanova S., Ryschich E., Mosl B. (2015). Prognostic and predictive value of immunological parameters for chemoradioimmunotherapy in patients with pancreatic adenocarcinoma. *British Journal of Cancer*.

[23] Egberts J.-H., Cloosters V., Noack A. (2008). Anti-tumor necrosis factor therapy inhibits pancreatic tumor growth and metastasis. *Cancer Research*.

[24] Utaisincharoen P., Tangthawornchaikul N., Ubol S., Chaisuriya P., Sirisinha S. (2000). Tnf-*α* induces caspase 3 (CPP 32) dependent apoptosis in human cholangiocarcinoma cell line. *Southeast Asian Journal of Tropical Medicine and Public Health*.

